# The expression landscape and pangenome of long non-coding RNA in the fungal wheat pathogen *Zymoseptoria tritici*

**DOI:** 10.1099/mgen.0.001136

**Published:** 2023-11-22

**Authors:** Hanna M. Glad, Sabina Moser Tralamazza, Daniel Croll

**Affiliations:** ^1^​ Laboratory of Evolutionary Genetics, Institute of Biology, University of Neuchâtel, 2000 Neuchâtel, Switzerland

**Keywords:** long non-coding RNA, *Zymoseptoria tritici*, expression analyses, pangenomes, plant pathogen

## Abstract

Long non-coding RNAs (lncRNAs) are regulatory molecules interacting in a wide array of biological processes. lncRNAs in fungal pathogens can be responsive to stress and play roles in regulating growth and nutrient acquisition. Recent evidence suggests that lncRNAs may also play roles in virulence, such as regulating pathogenicity-associated enzymes and on-host reproductive cycles. Despite the importance of lncRNAs, only a few model fungi have well-documented inventories of lncRNA. In this study, we apply a recent computational pipeline to predict high-confidence lncRNA candidates in *Zymoseptoria tritici,* an important global pathogen of wheat impacting global food production. We analyse genomic features of lncRNAs and the most likely associated processes through analyses of expression over a host infection cycle. We find that lncRNAs are frequently expressed during early infection, before the switch to necrotrophic growth. They are mostly located in facultative heterochromatic regions, which are known to contain many genes associated with pathogenicity. Furthermore, we find that lncRNAs are frequently co-expressed with genes that may be involved in responding to host defence signals, such as oxidative stress. Finally, we assess pangenome features of lncRNAs using four additional reference-quality genomes. We find evidence that the repertoire of expressed lncRNAs varies substantially between individuals, even though lncRNA loci tend to be shared at the genomic level. Overall, this study provides a repertoire and putative functions of lncRNAs in *Z. tritici* enabling future molecular genetics and functional analyses in an important pathogen.

## Data Summary

All datasets analysed in this study were accessed from NCBI (https://www.ncbi.nlm.nih.gov) with the following accession numbers. RNA-seq data for Zt09: PRJNA415716; RNA-seq data for 1A5, 1E4, 3D1 and 3D7: PRJNA327013; reference genomes 1A5, 1E4, 3D1 and 3D7: PRJEB15648, PRJEB20900, PRJEB20899, PRJEB14341; ChIP-seq data for IPO323: SRP059394.

**Impact Statement**

Long non-coding RNAs (lncRNAs) serve distinct roles from mRNA. Despite not encoding proteins, lncRNAs can control important cellular processes such as growth and response to stress. In fungal pathogens, lncRNAs are particularly interesting because they can influence how pathogens infect and harm their hosts. Yet, only very few fungal pathogens have high-quality repertoires of lncRNA established. Here, we use a recent computational method to identify lncRNA in the major wheat pathogen *Zymoseptoria tritici*. We found that lncRNAs are highly active during the early stages of infection, before the pathogen switches to necrotrophic growth. These lncRNAs are mainly located in regions of the genome associated with pathogenicity. The repertoire of expressed lncRNAs varies substantially among individuals, highlighting the potential for pathogen adaptation based on variation in lncRNAs. By expanding our knowledge of lncRNAs in important pathogen models, we enable future research to comprehensively investigate their roles across fungi.

## Introduction

Long-non-coding RNAs (lncRNAs) are a class of regulatory non-coding RNA (ncRNA) that lack a conserved set of defining features other than a length of over 200 nt, and the absence of the potential to encode a functional protein [1]. As RNA molecules, lncRNAs can interact with DNA, other RNAs and proteins to regulate a wide array of molecular processes at the transcriptional, post-transcriptional and translational levels. lncRNAs can influence the expression of genes through the recruitment of transcription factors and chromatin remodelling proteins, or through transcriptional interference [2–5]. lncRNAs may also influence the stability of a target mRNA or impact how a transcript is spliced [6]. Alternatively, lncRNAs can act as micro-RNA sponges or interact with ribosomes during translation [3]. The diversity of mechanisms by which lncRNAs can function is mirrored by the number of biological processes in which they intervene. In humans, many lncRNAs are differentially expressed in cancerous tissues, indicating their importance for DNA damage repair, genome stability and the regulation of autophagy [7–9]. A notable example is MALAT1 with an unusual up-regulation in cancer cells serving as a robust biomarker for several types of cancer including in breast and lung tissue [10, 11]. lncRNAs are also important in human immunity with several loci being strongly responsive to inflammation [3, 12, 13]. SNPs in lncRNAs are also associated with various immune-related diseases such as coeliac disease and atherosclerosis [14, 15]. lncRNAs tend to be more responsive to stress conditions than mRNAs [16, 17] in most organisms. In plants, lncRNAs are known to be important for the response to environmental stimuli. For example, ISP1 helps to maintain homeostasis during phosphate starvation [18, 19], and COOLAIR is an essential regulator of vernalization [19]. Naturally occurring variants of COOLAIR in populations of *Arabidopsis thaliana* require different lengths of cold exposure to de-repress flowering in early spring, and probably contribute to local adaptation [19–21]. Some lncRNAs probably contribute to the response to infection by a pathogen, through the regulation of resistance genes [22].

Interest in lncRNAs has greatly increased over the past two decades [23]. lncRNAs display higher sequence divergence and lower levels of inter-species conservation than protein-coding genes [24, 25]. Hence, lncRNAs are sometimes thought to be by-products of spurious transcription lacking any particular function [25]. Moreover, lncRNAs are typically expressed at low abundance compared to mRNAs, and often in a highly specific condition or cell-dependent manner [26], which makes their identification from sequencing data technically challenging [26, 27]. While lncRNAs can be transcribed from intergenic regions (lincRNA), lncRNAs are frequently intronic or antisense (lncNAT) to a protein-coding gene, complicating both the identification and functional validation, as knockout mutations are likely to impact not only the lncRNA but also the associated gene [28].

Most of our knowledge about the characteristics and functions of lncRNAs stems from well-studied model organisms [29]. However, recently, the functions of lncRNAs across a wider range of species, including pathogenic fungi, are being explored. As in plants and animals, lncRNAs in fungi are highly responsive to environmental conditions [30, 31], and can be important regulators of growth, reproduction and DNA damage repair. These processes are essential to any organism, but their fine-tuned regulation can be particularly critical for pathogens, which frequently encounter a wide variety of stressful conditions and must time reproductive cycles using signals from both the environment and the host. For example, the switch from yeast to hyphal growth before sexual reproduction is an important component of virulence for *Cryptococcus neoformans* and it has been shown that the main transcription factor responsible for orchestrating cellular differentiation, ZnF2, is itself regulated by an upstream lncRNA, RZE1 [32]. In the protist pathogen *Cryptosporidium parvuum,* the alternation between sexual and asexual reproductive phases is essential for host colonization, and 86 % of all predicted lncRNA candidates show differential expression between these two phases [33]. In *Candida auris*, the deactivation of the lncRNA DINOR results in higher levels of DNA damage and constitutive filamentous growth, demonstrating its role in maintaining genome integrity in stress conditions, such as during exposure to antifungal drugs [34]. In fact, fungal lncRNAs are frequently associated with the stress response, which is often a determinant of virulence as pathogens must survive the hostile environments created by host defence mechanisms. Predicted lncRNAs in the insect pathogen *Metarhizium robertsii* show high levels of differential expression during heat stress, with many predicted targets being directly implicated in responding to heat stress signals [31], and in *Candida*, many lncRNAs are differentially expressed during the infection of epithelial cells [34]. In *Ustilaginoidea virens*, fine-tuned transcription of the lncRNA UvlncNAT-MFS is directly required for conidiation and growth under stress [35].

Importantly, recent discoveries show that lncRNAs in fungal pathogens can have direct effects on virulence, for example through the regulation of enzymatic activity. Including in *Trichoderma reesei,* natural variants in the lncRNA HAX1 influence cellulase production [36], and in *Verticilium dahliae*, three lncRNAs were found to regulate the expression of cell-wall-degrading enzymes, with mutants resulting in decreased virulence on cotton [37] In *Cryptococcus neoformans,* lncRNAs were found in extracellular vesicles containing virulence factors known to modulate the host immune response [38], and in *Fusarium graminearum*, the lncRNA lncRsp1 influences virulence on wheat by regulating *Fgsp1*, which is required for normal ascospore discharge [39]. These examples illustrate the multitude of roles lncRNAs can play in pathogenicity-associated processes of filamentous fungi.

*Zymoseptoria tritici* is a filamentous ascomycete and the causal agent of Septoria blotch, one of the most detrimental crop diseases worldwide [40]. *Z. tritici* populations show high levels of genetic diversity even at small geographical scales [41, 42], coupled with significant variability in gene expression between isolates [43, 44]. The genome contains a high number of transposable elements (TEs) [45, 46] which may provide frequent opportunities for the formation of functional lncRNAs [47]. During infection on wheat, *Z. tritici* undergoes a switch from a biotrophic to necrotrophic lifestyle, and a vast transcriptional reprogramming is required for the necessary metabolic changes [43, 44]. Significant morphological changes have also been observed during growth in stressful conditions [48]. Epigenetic control of TE-rich, accessory regions probably contributes to high levels of expression variation of infection-related genes [49]. An increase in TE expression and an enrichment of small RNAs (sRNAs) originating from accessory chromosomes was observed during growth in nutrient-poor conditions, indicating a potential role of these regions in the response to stress [50]. Despite a clear transition in the sRNA transcriptome under stressful conditions, no direct role in host colonization has been demonstrated for sRNAs, in contrast to several other fungal plant pathosystems [51]. Recently, an improved genome annotation based on long-read transcript sequencing was established [52] reporting the production of several lncRNAs during growth *in vitro*. The study further demonstrated that lncRNAs were differentially expressed between *in vitro* and *in planta* conditions, and that some lncRNAs showed interesting expression correlation patterns with nearby genes during infection [52].

In this study, we identify and assess high-confidence candidates to reveal the landscape of lncRNAs in the *Z. tritici* genome, identify biological processes associated with lncRNA functions and assess expression variation over an infection life cycle. Finally, we assess pangenome features of lncRNA loci to define conserved and variable elements of the genomic and transcriptomic landscape of lncRNA.

## Methods

### lncRNA candidate identification

Raw RNA sequencing (RNAseq) reads from the *Z. tritici* reference isolate Zt_09 (IPO323ΔChr18) produced at four stages of the infection cycle *in planta* were downloaded from NCBI (accession PRJNA415716) [44]. Using a machine-learning-based ncA prediction tool, PINC [53], we combined these data with the reference genome annotation for IPO323 [54] to obtain a list of predicted lncRNA candidates. The weight of the Youden’s index was raised from 0.5 to 0.7, in order to decrease false-positive rates [53]. We included consensus sequences of annotated TEs [45] in the file containing known protein-coding mRNAs in order to reduce the number of predicted transcripts originating from degraded TE insertions. We retained only the longest predicted transcript at each locus. The resulting annotation containing predicted lncRNAs was compared using gffcompare (v0.11.2) [55] to the reference annotation for IPO323.

### Differential expression and expression variation analysis

RNAseq reads were aligned to the reference genome IPO323 using Hisat2 [56] with default parameters and exon-level counts were quantified with featureCounts (v2.0.1) [57] using default parameters. FeatureCounts was run twice: once using the GTF file provided by the output of PINC and once using the reference annotation, so that we could more accurately quantify expression of reference transcripts that were missing in the stringTie (v2.2.1) [58] assembly performed within PINC. lncRNAs and mRNAs were tested for differential expression between early and late infection stages by grouping counts for the two earliest time points and testing against the two latest. Tests were performed using the *edgeR* package (v3.17) [59] in R (v4.3.1) [60] with a false discovery rate (FDR) cut-off of 5 %. To compare expression between isolates, reads from four additional isolates (i.e. 1A5, 1E4, 3D1 and 3D7; accession PRJNA327013) were downloaded from NCBI and aligned to each respective reference-quality genome (accessions PRJEB15648, PRJEB20900, PRJEB20899 and PRJEB14341) [61, 62] using Hisat2 with default parameters. Mapped reads were quantified with featureCounts using the GTF file containing the PINC predictions. The expression variation of a transcript at a given time point was defined as the coefficient of variation in Trimmed Mean of *M*-value (TMM) counts, calculated between all isolates. All transcripts with <10 c.p.m. in one or more of the three biological replicates for each isolate and each time point were removed, in order to reduce noise originating from lowly expressed loci.

Clustering TMMs of protein-coding genes and lncRNAs in the reference isolate Zt09 were scaled to achieve comparable variance across the four time points. Values of both mRNAs and lncRNAs were clustered simultaneously according to their expression trajectories across the time points using fuzzy c-means clustering implemented in the *e1071* (v1.7-13) [63] R package. Helper functions [64] were used to define optimal hyper-parameters. Within-sum-of squares indicated that the optimal number of clusters was 7. After clustering was performed, clusters were qualitatively grouped into three groups based on peak expression: early expression peak (first two time points), late expression peak (last two time points), and bi-modal expression peak (showing high expression at both the earliest and latest time points with reduced expression in between). All protein-coding genes belonging to each cluster were extracted and used as groups of interest for functional enrichment analysis. All analyses were performed in R.

### Functional enrichments

Functional domains for all reference genes were extracted from a previous annotation [54]. All enrichment analyses were performed using the *Gostats* package (v3.17) [65] in R, by comparing the annotated functions of genes in a group of interest (e.g. expression clusters) to the functions of all genes in the reference genome. Target prediction and characterization, expression correlation, and secondary structure predictionAll transcripts within 50 kb of an lncRNA were extracted from the reference genome using bedtools (v2.30.0) [66]. lncTAR [67] was used to predict potential RNA interactions between lncRNA/mRNA pairs in this window. Pairs were considered to have a potential interaction if the normalized binding free energy (ndG) was lower than the default cut-off (−0.1). The expression (centred and scaled TMMs) of each transcript in the window was correlated (Pearson’s *r*) to the expression of the corresponding lncRNA, and the distribution of correlation coefficients of interacting pairs was compared to non-interacting pairs at different distances. For selected lncRNAs, RNAfold was used to predict secondary structure, using default parameters. Functional domains encoded by selected interacting genes were extracted from previous annotations [54]. Biosynthetic gene clusters were predicted using antiSMASH v.7.0 [68].

### Chromatin immunoprecipitation sequencing (ChIP-seq)

We compared histone modification H3K27me3 and H3K4me2 profiles of the reference genome of *Z. tritici* (IPO323) cultured in carbon-limited medium (minimal medium) and carbon-rich medium [yeast-sucrose broth (YSB)]. We performed culturing for ChIP-seq analyses of *Z. tritici* based on growth in Vogel’s Medium N (minimal medium) until hyphae formation for 8 days at 18 °C. The ChiP-seq library was prepared for sequencing and analysed using a NovaSeq 6000 in paired-end mode with a read length of 150 bp. ChIP-seq data of *Z. tritici* grown in YSB medium were retrieved from the NCBI SRA database (accession number SRP059394); ChIP-seq raw reads were trimmed using Trimmomatic v.0.32 [69] with parameters ILLUMINACLIP:2 : 30 : 10 LEADING:3 TRAILING:3 SLIDINGWINDOW:4 : 15 MINLEN:36, and mapped to the reference genome IPO323 with Bowtie2 v.2.4 [70] --very-sensitive-local parameter. Duplicated reads were tagged with the GATK Picard MarkDuplicates function v.4.2.4.1 . Peak calling was performed with Epic2 v.0.0.52 [71] setting bin-size 1000 –mapq 5.

### Pangenome analyses

Fasta sequences of predicted lncRNAs were extracted from the transcriptome assembly output by PINC using bedtools, and were aligned to the genome sequences of 18 alternative reference isolates using exonerate (v2.70.2) [72] (model est2genome with a maximum intron length of 300). Transcripts were considered present in a genome as long as they returned a significant match. Transcripts present in at least 18 of the 19 isolates were classified as core, while the rest were classified as accessory. We did not distinguish between soft core (typically 95 % of isolates) and core transcripts (99 % of isolates), on the one hand to retain consistency with the previous pangenome structure defined for proteins [73] and on the other because both categories round to the same lower bound in our dataset (i.e. 18 isolates). To estimate the number of additional lncRNAs that could be present on a population scale, the steps outlined in candidate identification were repeated for four other isolates with available transcriptomic data (1A5, 1E4, 3D1 and 3D7). The resulting transcript sequences were clustered with cd-hit (v4.8.1) [74] using relatively relaxed parameters (global sequence identity ≥80 %, minimum reciprocal alignment coverage ≥75 %), as lncRNAs may diverge more rapidly than protein-coding transcripts. Transcripts belonging to the same cluster were considered as the same lncRNA regardless of genomic location or context. The accumulation curve was constructed by treating the clusters of transcripts as orthogroups, using the *vegan* library (v2.6-4) [75] in R. Genomes were sampled without replacement. Correlation plots were created using the corrplot package in R [76]. All additional statistical tests were also performed in R and all plots were created using the ggplot2 package v3.4.2 [77].

## Results

### Identification and characterization of lncRNA candidates across the genome

We predicted lncRNA loci based on transcriptomic datasets collected across multiple environments. RNAseq data from the *Z. tritici* reference isolate Zt09 generated at four stages of the infection cycle on wheat plants were mapped to the reference genome IPO323 [44]. Zt09 is a derivative (IPO323ΔChr18) of the reference strain differing only by the deletion of the accessory chromosome 18 [41]. We used a machine-learning-based prediction pipeline, PINC [53], to predict lncRNA candidates, integrating the consensus sequences of all known TEs as well as all annotated genes as known mRNA sequences. This reduced the number of predicted lncRNAs originating from TEs. We obtained a total of 120 putative lncRNA candidates originating from 108 distinct loci. Of the 12 loci predicted to produce multiple lncRNAs, six were predicted to produce more than one transcript of exactly the same length. While lncRNA isoforms are known to exist in other organisms [78], isoforms are not expected to show the same transcription start and stop site, as well as be of exactly the same length. Furthermore, RNA-isoform-level downstream analysis requires care and cannot be fully resolved using single-stranded RNA-seq data [79]. In order to facilitate downstream analysis and avoid false calls originating from transcript assembly, we filtered the original lncRNA candidates to retain only the longest transcript at each locus. This resulted in a total of 91 predicted lncRNAs (Table S1, available in the online version of this article).

A revised IPO323 genome annotation based on culture medium Iso-Seq libraries predicted 51 lncRNAs [52]. Only two of our 91 lncRNA (2.2%) candidates overlap with these predictions. Low congruence between these two pipelines may be a result of several factors: the authors excluded all transcripts under <1 kb and containing an ORF longer than 300 bp [52], which differed from our pipeline that uses two machine-learning models to assess coding potential in relation to known coding sequences instead of filtering by ORF length [52]. Furthermore, repertoires of expressed lncRNA may be significantly different between *in vitro* and *in planta* conditions. Although the majority of our candidates were not predicted to be lncRNA in this new annotation, our candidates were generally supported by the Iso-Seq data. Despite being only from culture medium condition, a total of 62.8 % of our candidates have at least one fully covering read, and 78.26 % show non-zero coverage over more than 75 % of their length (Fig. S1).

**Fig. 1.**
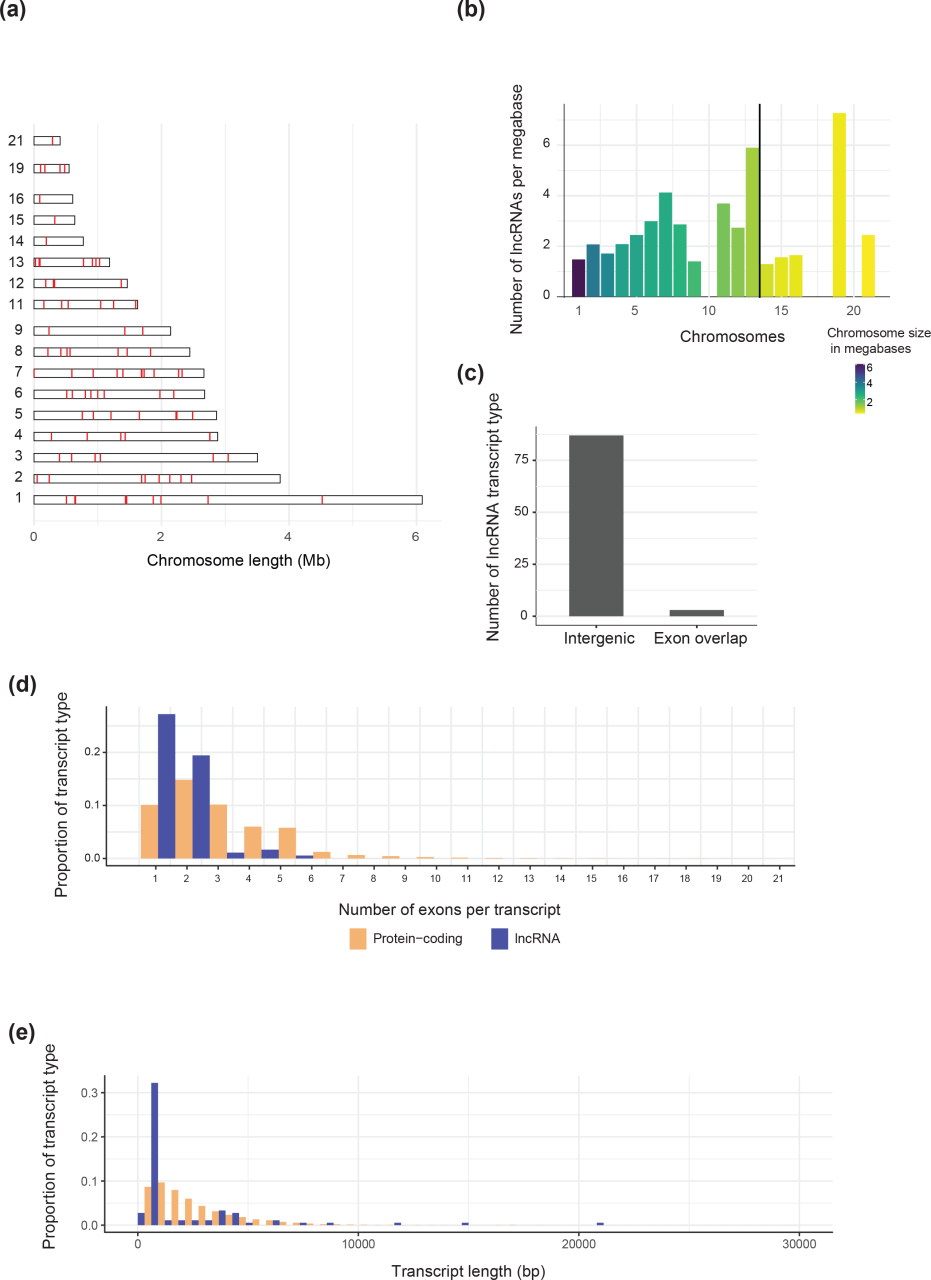
(a) Schematic representation of lncRNA loci distributed along chromosomes. Each red line represents a predicted lncRNA. (**b**) Density of lncRNAs on each chromosome. (**c**) Bar chart showing the number of intergenic (no overlap with a known coding sequence) versus gene overlapping (overlapping at least one exon) lncRNAs. The black line separates core chromosomes (left) and accessory chromosomes (right). (**d**) Histogram comparing the number of exons per transcript between lncRNAs and mRNAs. (**e**) Histogram comparing the transcript length in base pairs between lncRNA and mRNA.

lncRNA loci were found on most chromosomes, with the exception of chromosomes 10, 16, 17 and 20 (Table S1). Chromosomes carried 1–13 lncRNAs with the highest number found on chromosome 7. The large majority (91.1%) of lncRNAs were located on core chromosomes (excluding chromosome 10). The maximum number of lncRNAs found on an accessory chromosome was four (chromosome 19) (Fig. S2). The distribution of lncRNAs along the chromosomes showed no apparent associations with chromosomal features, and loci were discovered both in non-telomeric and in sub-telomeric regions (Fig. 1a). The density of lncRNA was largely dependent on the total size of the chromosome, except for chromosome 7, which despite a relatively large size displayed not only the highest number of lncRNAs but also the third highest density (Fig. 1b). Of the 91 lncRNAs, 87 were classified as lincRNAs while only three were lncNATs (Fig. 1c). Compared to genes, lncRNA loci encode in general fewer exons (Fig. 1d) (mostly one or two) and transcripts were slightly shorter on average (Fig. 1e), which is consistent with knowledge about lncRNAs in other organisms [23].

### Genomic niches and expression dynamics of lncRNA loci

**Fig. 2.**
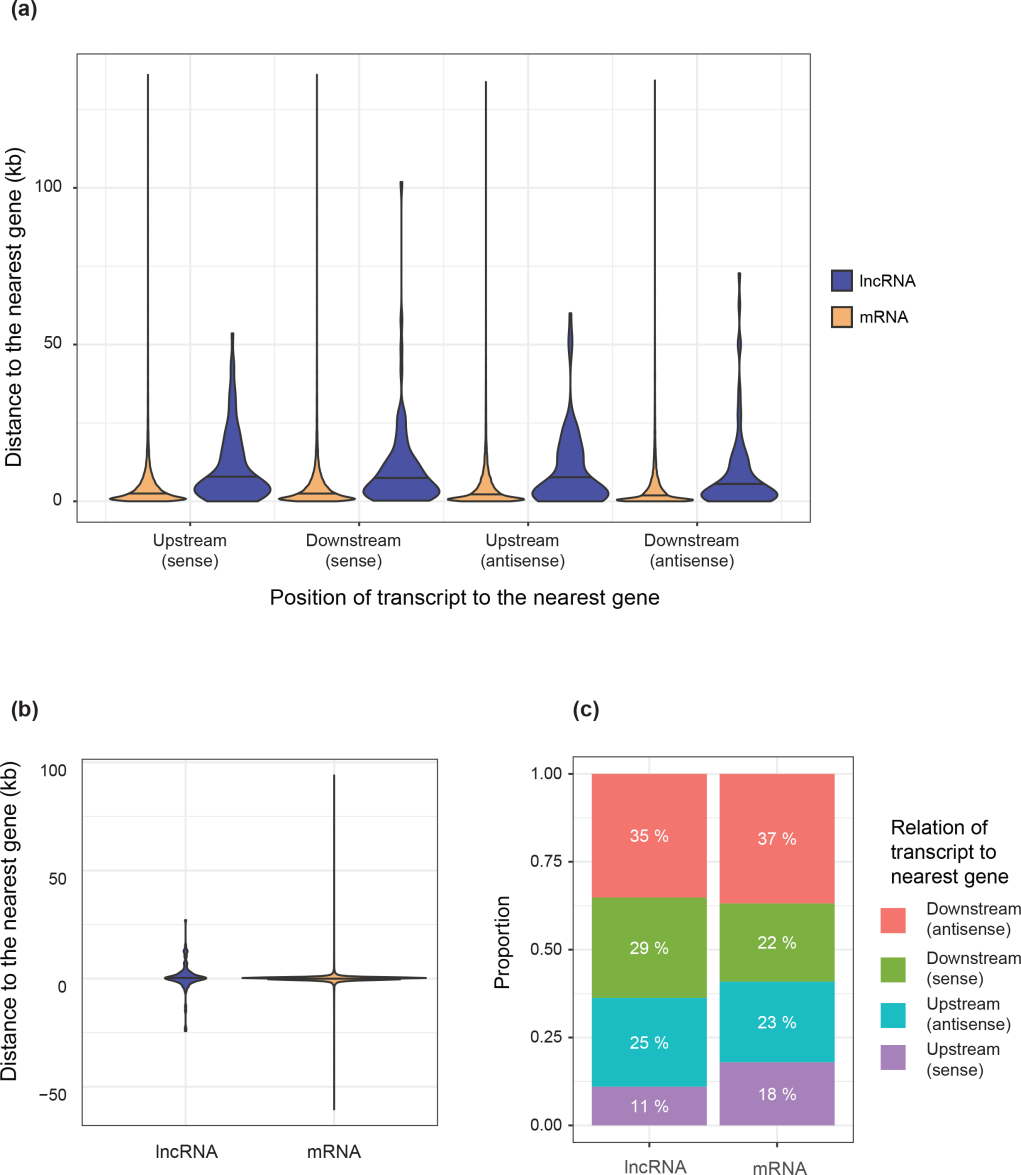
(a) Violin plot comparing distances to the nearest neighbouring gene in each orientation for lncRNA and mRNA. Upstream and downstream refer to the position of the lncRNA or mRNA relative to the neighbour. Sense and antisense refer to whether the lncRNA or mRNA is transcribed from the same strand as the neighbour. (**b**) Violin plot comparing the distance to the nearest neighbouring gene, regardless of orientation. (**c**) Comparison of the orientation of the nearest neighbouring gene between lncRNAs and mRNAs.

To compare the genomic context of lncRNAs to that of protein-coding genes, we assessed the distance of each lncRNA to its nearest neighbouring genes. Compared to protein-coding genes, lncRNAs showed no differences in the distance to the nearest gene in any individual orientation (sense upstream/downstream, antisense upstream/downstream; Fig. 2a). Moreover, there was no significant difference in the distance to their nearest neighbour regardless of orientation (Fig. 2b). Next, we assessed if lncRNAs were more likely to be found in a particular orientation relative to their nearest neighbouring gene (Fig. 2c). We found no significant differences between lncRNAs and protein-coding genes (chi-squared test; χ^2^=2.8395, d.f.=3, *P*=0.417). Taken together, these results indicate that lncRNAs are found in similar genetic contexts as protein-coding genes.

**Fig. 3.**
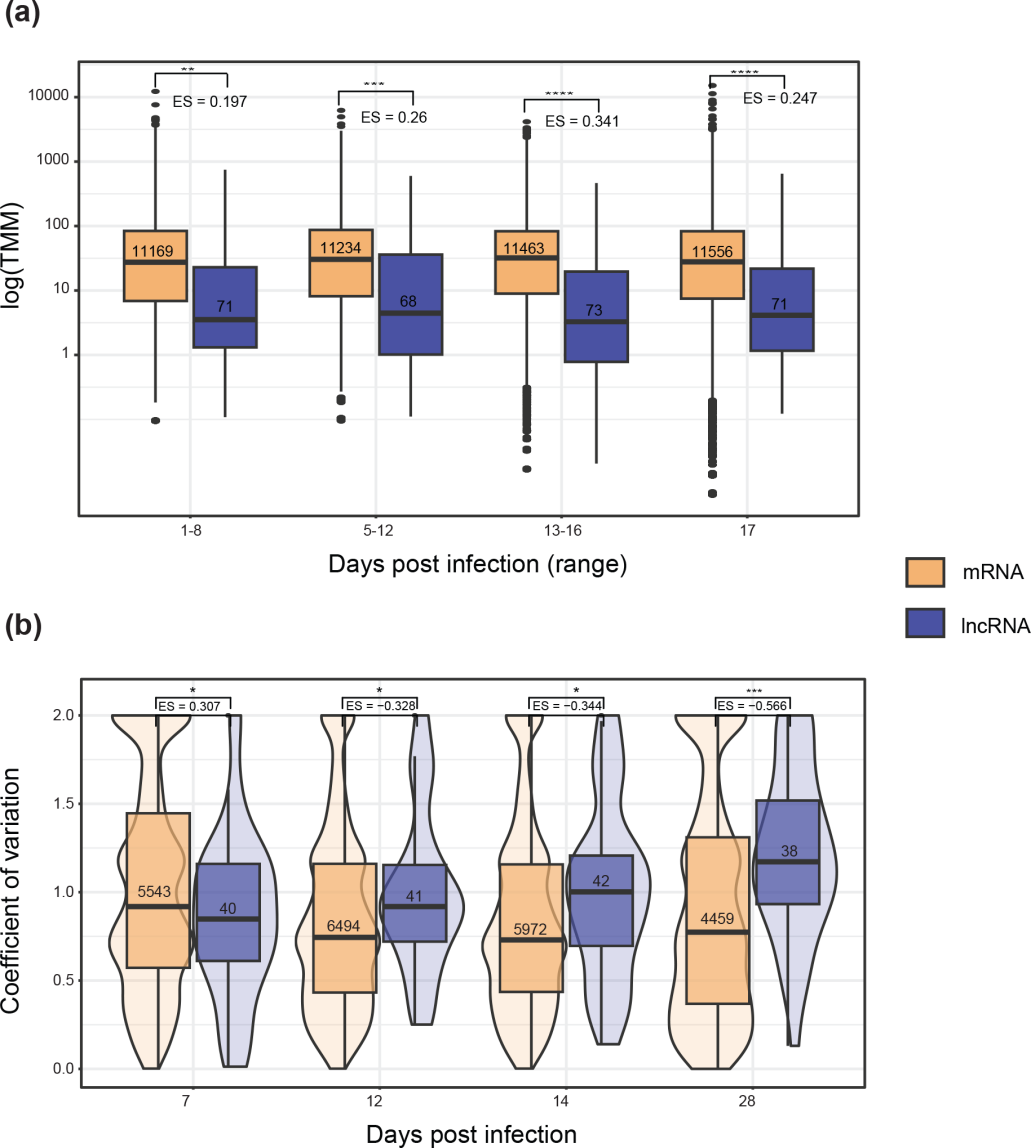
(a) Box-plots comparing log-TMM values between lncRNA and mRNA across the infection cycle. Stars show the significance of Welch's two-sided *t-*tests comparing the mean expression of each transcript type at each time point. Values underneath the brackets (denoted by ‘ES=”) show the effect size relative to mRNA expression. Values inside the boxes show sample size. (**b**) Violin plot comparing tthe coefficient of variation (CV) of expression (log-TMM) for each individual transcript across four isolates, at each time point. Stars show the significance levels of Welch's two-sided *t-*tests comparing the mean CV between lncRNAs and mRNAs. Values denoted by ‘ES=’ represent the effect size relative to the mean mRNA CV at each time point.

lncRNAs are known to be expressed at lower levels than mRNAs [23]. To test this in *Z. tritici*, we assessed TMMs by infection time point for both lncRNAs and mRNAs. As expected, lncRNAs were significantly less expressed at all four time points capturing the infection lifestyle transitions with an average effect size of −2.6 compared to protein-coding genes (Fig. 3a). lncRNA and mRNA expression differences were assessed in four additional strains with comparable RNA-seq datasets. As in the reference strain, lncRNAs were expressed at consistently lower levels compared to mRNA across all isolates (Fig. S3). Intraspecific variation in lncRNA expression is known to be higher than for protein-coding genes across kingdoms [16, 80, 81]. To test for such differences, we compared the expression variation of lncRNA to mRNA by calculating the coefficient of variation for each transcript using four additional strains, after removing lowly expressed transcripts to reduce noise. With the exception of the first time point at 7 days post-infection (dpi), lncRNAs show a significantly higher variability in expression than mRNAs at all time points, with the strongest effect at 28 dpi (Fig. 3b).

### Potential regulatory targets of lncRNA

Regulatory effects of most known lncRNAs are acting *in cis* with a minority acting *in trans* [82]. However, targets *in cis* are also computationally less demanding to detect, given the smaller search space for associations across the genome. To identify the range of potential *cis* targets of the identified lncRNAs, we extracted all annotated genes within a 50 kb window around each lncRNA-encoding locus. Given the average distance between genes of ~1 kb, the window typically contains dozens of genes. Compared to the rest of the genome, genes within the window are enriched for molecular functions involving catalytic and enzymatic activity as well as binding functions (most significantly ATP and lipid binding) (Fig. S4). In terms of biological processes, genes close to lncRNAs are enriched in functions related to protein metabolism and modification processes (Fig. S5).

**Fig. 4.**
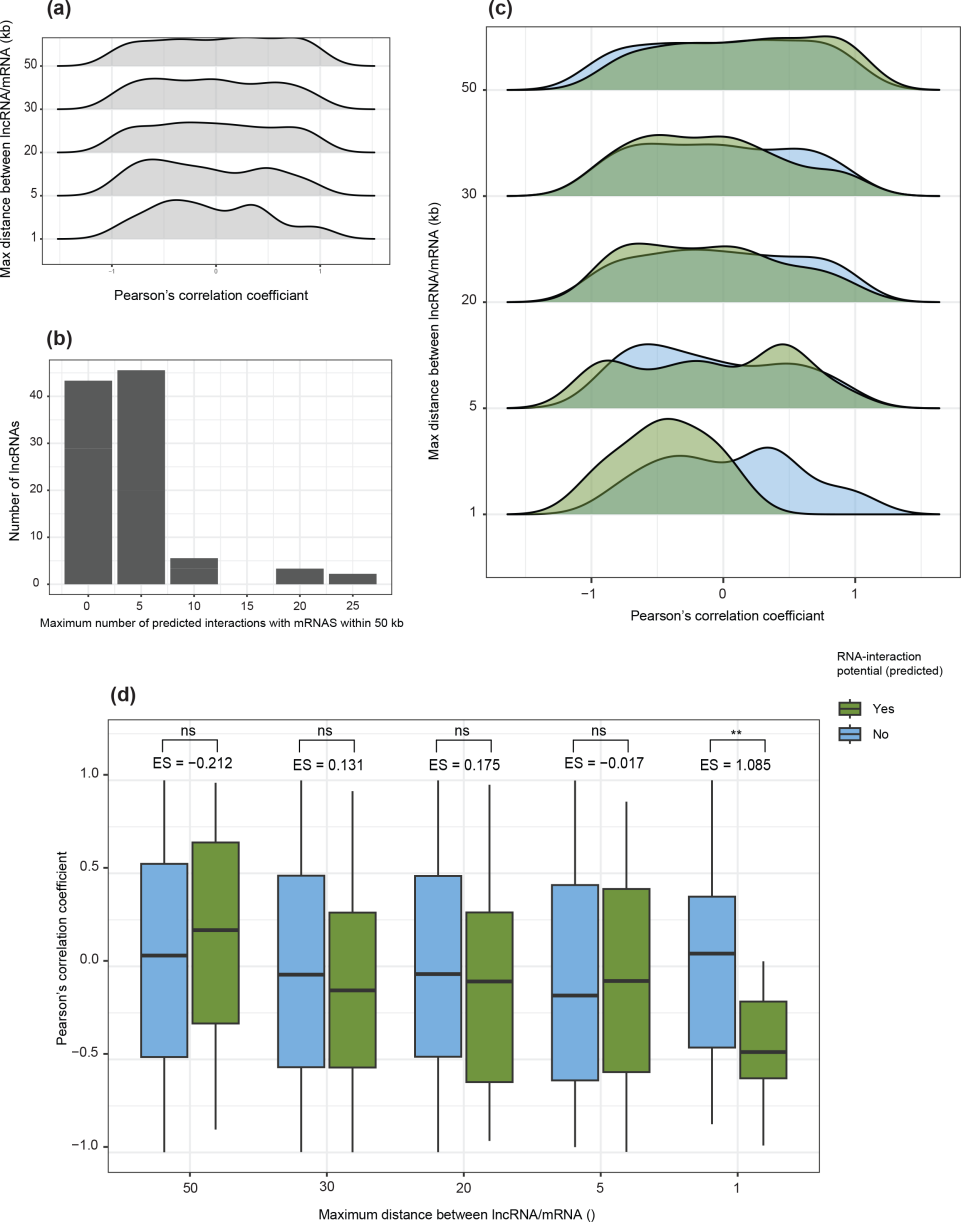
(a) Density plots comparing correlation coefficients (Pearson’s *r*) for expression values (log-TMM) between pairs of lncRNA and mRNA, grouped based on the distance in base pairs between the pair. The maximum distance separating the pairs is represented on the *y*-axis, with the minimum being the boundary of the neighbouring group; for example, the distribution shown at 50 kb represents all lncRNA–mRNA pairs between 30 and 50 kb. (**b**) Total number of significant lncRNA–mRNA interactions per lncRNA, predicted by free energy minimization. (**c**) Density plots comparing correlation coefficients (Pearson’s *r*) for expression values based on the distance between the pair and their potential to interact. Pairs with no predicted interaction are shown in blue, while pairs with a predicted interaction are shown in green. (**d**) Boxplots comparing correlation coefficients (Pearson’s *r*) for expression values for pairs of lncRNAs and mRNAs based on the distance between the pair and their potential to interact. Pairs with no predicted interaction are shown in blue, while pairs with a predicted interaction are shown in green. Stars represent the significance levels of Welch's two-sided *t-*tests comparing the mean expression correlation coefficient of interacting and non-interacting pairs at each distance. Values denoted by ‘ES=’ show the effect size relative to the correlation coefficients of non-interacting pairs.

Proximity to genes is insufficient to ascertain a *cis-*acting lncRNA function, and hence we assessed whether the expression of protein-coding genes and potential lncRNA regulators were positively or negatively correlated for all pairs within a range of 50 kb. Overall, no trend in strength or direction of correlations were found based on the distance of an lncRNA to an mRNA. Additionally, no differences were observed between correlation coefficients of lncRNAs and mRNAs within 50 kb compared to random pairs of genes, indicating that proximity alone does not drive lncRNA–mRNA co-expression (Fig. 4a; Table S2). lncRNAs may modulate the expression of nearby genes by interacting with an mRNA to form RNA–RNA duplexes [83, 84], and the potential for two RNAs to interact can be predicted by free-energy minimization [85]. Using the software lncTAR [67], we analysed the potential interactions between lncRNA and mRNAs in the same 50 kb windows. We found that 43.3 % of lncRNAs had no predicted RNA interaction, while 45.6 % of lncRNAs had predicted interactions with 1–5 mRNAs. The maximum number of predicted lncRNA–mRNA interactions in a 50 kb window was 23 (Fig. 4b). We compared expression correlations between interacting and non-interacting pairs (Fig. 4c). We found that pairs of lncRNAs and mRNAs that could interact were significantly more anti-correlated with each other than pairs that were not predicted to interact, as long as the lncRNA–mRNA pair was located within 1 kb (effect size 1.085) (Fig. 4d). At larger distances, the potential for the transcripts to interact was not significantly associated with expression correlation (Fig. 4d).

To investigate gene functions and pathways potentially regulated by lncRNAs, we performed an enrichment analysis of all predicted mRNA targets at a maximum distance of 5 kb and showing an absolute correlation coefficient >0.5 with the interacting lncRNA against the genomic background. Most enriched biological processes were related to oxygen stress and detoxification (Fig. S6). The strongest enriched molecular function was antioxidant activity (Fig. 7). Among all potential lncRNA targets, regardless of distance or correlation, enriched biological pathways include regulatory processes involved in homeostasis, as well as translation, ion transportation and metabolism (Fig. 8). Metal-ion transmembrane transporter activity is the most strongly enriched molecular function among all the potential mRNA targets compared to the genome (Fig. S9), and it is also enriched when compared to non-target genes within the same 50 kb interval (Fig. S10).

### Differential expression of lncRNAs during plant infection

The infection cycle of *Z. tritici* on wheat includes four distinct morphological stages, characterized by the up-regulation of particular gene functions and pathways [43, 44, 86]. *Z. tritici* isolates are highly diverse both genetically and transcriptionally, and as a result the timing of each stage varies significantly between isolates [43, 44]. For simplicity, we refer here to the timing of each stage according to the reference isolate Zt09. At the earliest stages (1–8 dpi), spores germinate on leaves and hyphae enter through the stomata. From 5 to 12 dpi, the pathogen colonizes the mesophyll concluding the biotrophic stages. During biotrophic growth, genes involved in lipid catabolism are up-regulated, indicating that the pathogen is relying on internal energy storage [86]. The highest number of predicted effectors are up-regulated at the 5–12 dpi stage [86]. From 13 to 16 dpi, the pathogen forms pycnidia as it begins to acquire nutrients from the host and enters the necrotrophic stage, characterized by the up-regulation of cell-wall-degrading enzymes, transmembrane transporters, and genes involved in secondary metabolite production. Beyond ~17 dpi, the pathogen can produce pycnidia inside the stomatal cavity [44].

**Fig. 5.**
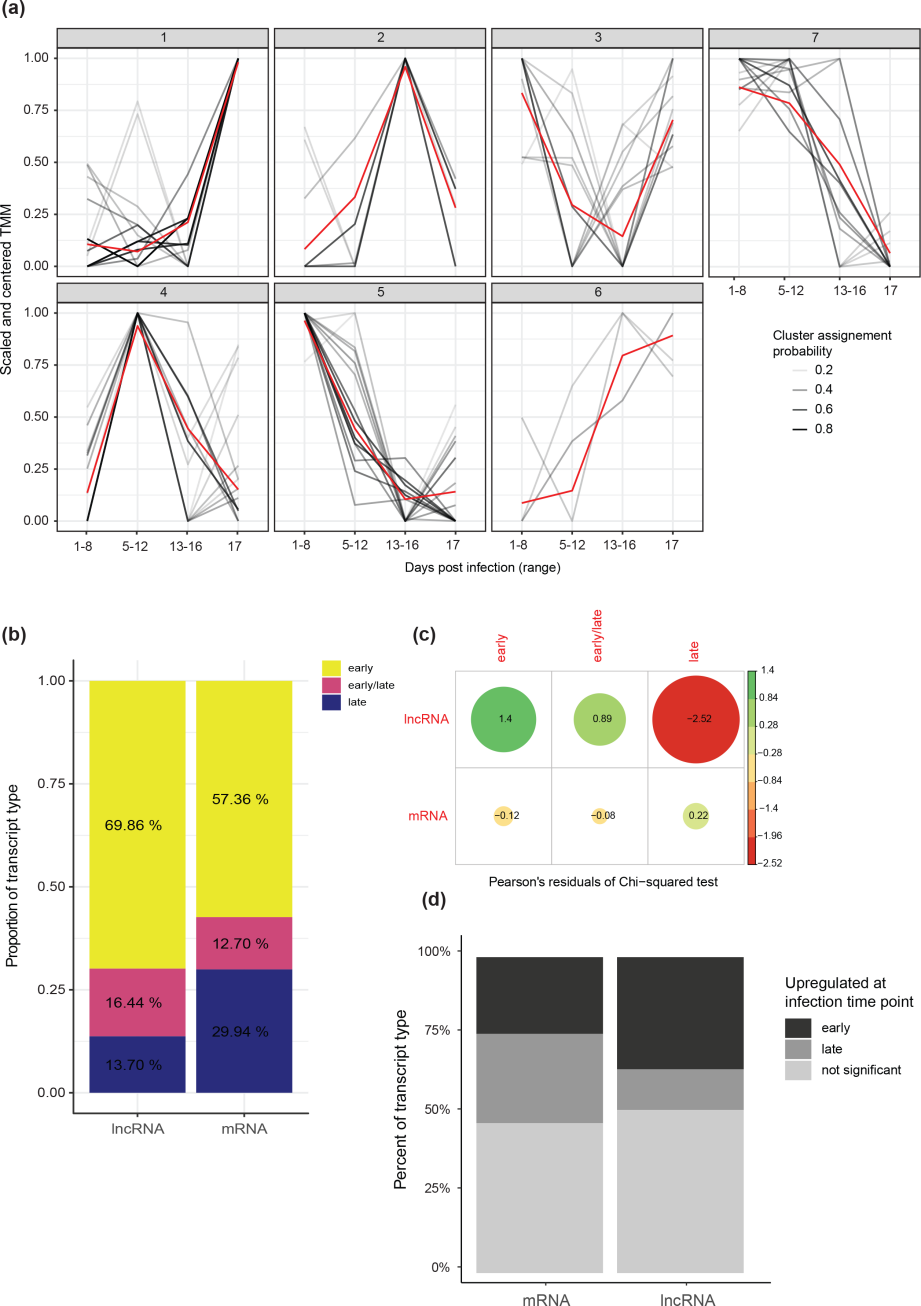
(a) Expression patterns of lncRNAs (black) across the infection cycle. Clusters were defined by fuzzy c-means, using the centred and scaled TMM values of both lncRNAs and mRNAs at each time point. The shade of the line shows the confidence of assignment of each lncRNA to the cluster, with darker lines having a higher confidence. (**b**) Distribution of lncRNAs and mRNAs into groups of expression clusters based on the peak expression of each cluster. Clusters in pink, defined as ‘early/late’, show bimodal peak expression, with high values during early and late infection, and lower values at intermediate time points. (c) Correlation plot showing the association between transcript type and cluster group. Dot size represents the importance of the association while colour indicates direction. (**d**) Bar chart comparing the number of transcripts that are differentially expressed during early infection, late infection and transcripts not differentially expressed between lncRNA and mRNA.

In order to understand the developmental context of lncRNA expression, we grouped all transcripts (both lncRNA and protein-coding) into expression clusters using fuzzy c-means clustering on their expression trajectories across the infection time points (Fig. 5a). Both protein-coding genes and lncRNAs were attributed to all seven clusters. Clusters with similar expression profiles were grouped to obtain three groups of clusters, one with peak expression during early infection, one during late infection, and one with high expression at both early and late time points (Fig. 5b). lncRNAs were more likely to show peak expression during early infection compared to protein coding genes (chi-squared test; *P*=0.01015; Fig. 5c). Clusters 4 and 5 contained the two highest numbers of lncRNAs and both showed peak expression during early infection. Cluster 5 includes the highest number of lncRNAs, and is enriched in genes encoding nucleic-acid-binding domains, involved in catalytic activity, and oxidoreductase/peroxidase/antioxidant activity (Fig. S11), which have been shown to be important for overcoming the host defence response activated upon infection [87, 88]. Notably, secreted peroxidases are important pathogenicity factors for *Z. tritici,* and are required for symptom formation on wheat [89]. Cluster 4 shows an enrichment of functions related to serine peptidases, sulphur transmembrane transporters and vitamin B6/pyridoxine binding (Fig. S12). Serine peptidases are involved in a number of essential intra- and extracellular functions, with roles both in nutrient acquisition and in immunity evasion [90, 91], and have been directly implicated in pathogenic interactions between fungi and various hosts, including plants. Sulphur metabolism is a core component of plant–pathogen interactions and plants secrete sulphur-rich molecules as a means of defence [92]. Vitamin B6/pyridoxine metabolism is linked to oxidative stress relevant for both plant defence and fungal pathogenicity [93, 94]. Serine hydrolase and antioxidant activities are among the significantly enriched functions of genes in the proximity of identified lncRNAs (Fig. S4). Regulation of hydrolase activity is the strongest enriched biological process among all potential targets compared to the rest of the genome (Fig. S8), and the response to oxidative stress was the strongest enriched biological process for targets showing strong correlation with their interacting lncRNA (Fig. S6).

We assessed probable functionality of lncRNAs by testing for differential expression between early infection (time points A+B corresponding to 1–12 dpi) and late infection (C+D or 12–17 dpi). Transcripts were filtered for ≥10 reads across the three replicates, retaining 62 lncRNAs. Nearly half (*n*=30) out of these were found to be differentially expressed between early and late time points (FDR 5%) (Table S3). We found only eight up-regulated lncRNAs compared to 22 down-regulated lncRNAs at the late infection stage (Fig. 5d). Of the 30 differentially expressed lncRNAs, 29 had an absolute correlation coefficient (Pearson’s *r*) >0.8 with at least one gene within 50 kb (Table S4). Moreover, 11 lncRNAs had at least one predicted interaction with a neighbouring mRNA, of which eight had a strong absolute (*r*>0.8) correlation coefficient with their predicted mRNA target (Table S5).

We examined the differentially expressed lncRNAs and identified two showing strong expression correlations and predicted RNA interactions with nearby genes. MSTRG.9312.1 is up-regulated during early infection and located ~3 kb away from a predicted RiPP-like biosynthetic gene cluster (RiPP: ribosomally synthesized and post-translationally modified peptide) that contains 26 genes. MSTRG.9312.1 (Fig. S13) is strongly correlated (*r*<−0.8; *r*>0.8) with six genes in this cluster (Table S6) and most significantly with *Zt09_7_00402* (*r*=0.965; *P*=0.034), from which the lncRNA is antisense downstream (Fig. S14). This gene encodes an uncharacterized protein with a DnaJ domain, typical of heat-shock proteins (HSPs), which are known to be implicated in various stress responses [95]. Additionally, MSTRG.9312.1 is predicted to be able to interact with the mRNA produced by the nearest same-sense neighbour, *Zt09_00404* (ndG=−0.1029) and is significantly positively correlated (*r*=0.961; *P*=0.038).

**Fig. 6.**
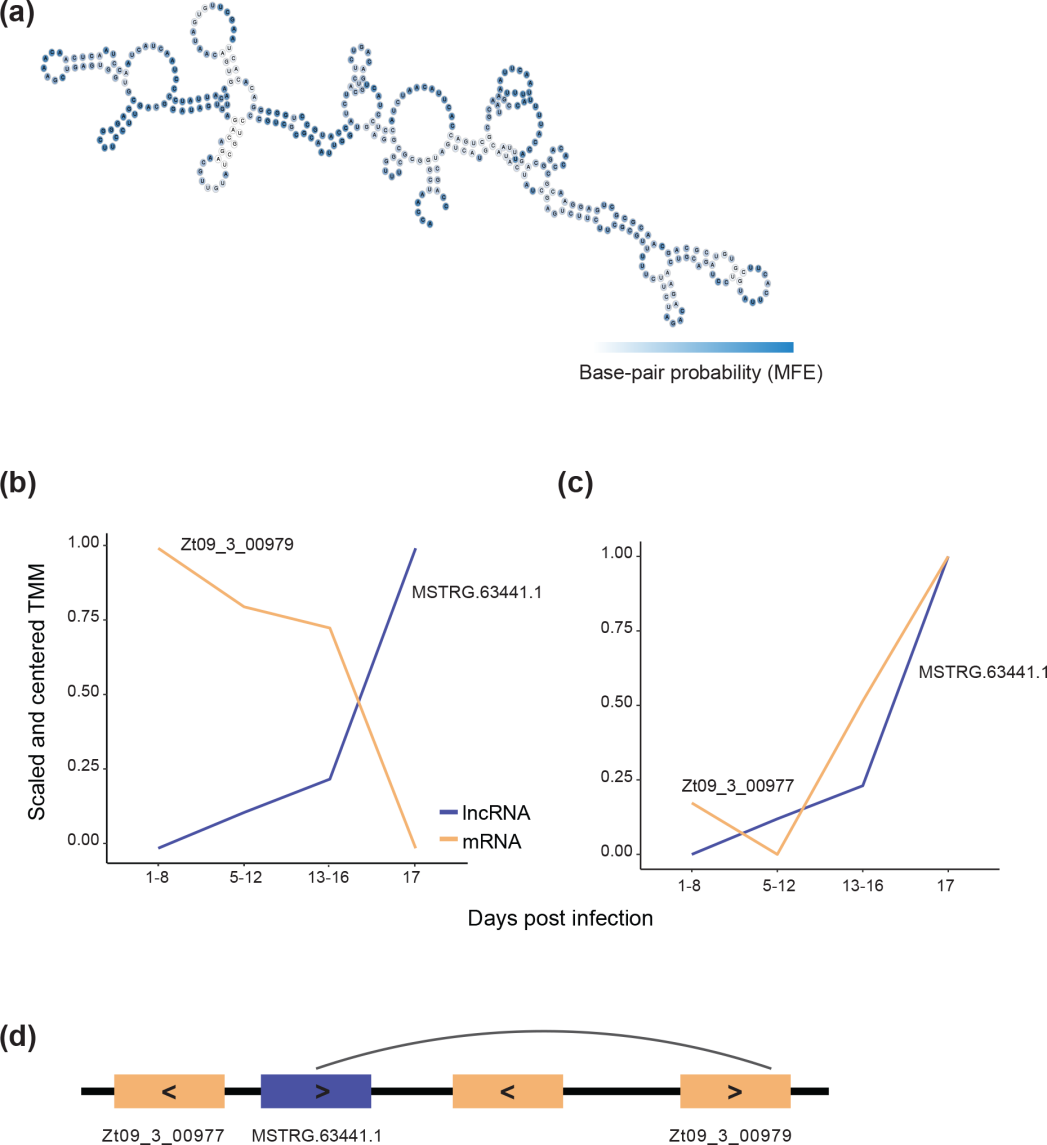
(a) Secondary structure of the lncRNA MSTRG.63441.1 as predicted by free energy-minimization. The colour shows the base pair probabilities. (**b**) Trajectories across the infection cycle of the lncRNA MSTRG. 63 441.1 (blue) and the nearby gene Zt09_3_00979 (orange). (**c**) Expression pattern across the infection cycle of the lncRNA MSTRG. 63 441.1 and the nearby gene Zt09_3_00977. (**d**) Schematic representation of the position of the lncRNA relative to the two genes. Arrows indicate the strand from which each element originates.

The lncRNA MSTRG.6344.1 shows strong up-regulation during late infection (log fold-change=2.08, *P*=0.03) and a strong negative correlation with the expression of the gene *Zt09_03_00979* (*r*=−0.997; *P*=0.0028) (Fig. 6a, b). *Zt09_03_00979* is one of the predicted mRNA targets of the lncRNA (ndG=−0.101) and is located ~5 kb upstream on the same strand (Table S7). *Zt09_3_00979* encodes an uncharacterized protein with an RNA recognition motif and a domain potentially interacting with the arginine methyl-transferase HMT1 (PRMT1 homologue), known to be implicated in chromatin dynamics through H4R3 methylation [96]. The activity of HMT1 on non-histone proteins has been shown to be important for virulence, growth and the response to stress in *F. graminearum* [97]. MSTRG.63441 is also strongly positively correlated, with *Zt09_03_00977* (*r*=0.91) (Fig. 6c) which encodes an uncharacterized protein with an OTT_1508-like deaminase domain. The gene is located ~700 bp upstream of the lncRNA transcribed from the opposite strand and in the opposite direction. The nearest upstream gene to the lncRNA is *Zt09_03_00978* with which it shows no transcriptional correlation (Fig. 6d).

### Epigenetic states of lncRNA loci

**Fig. 7.**
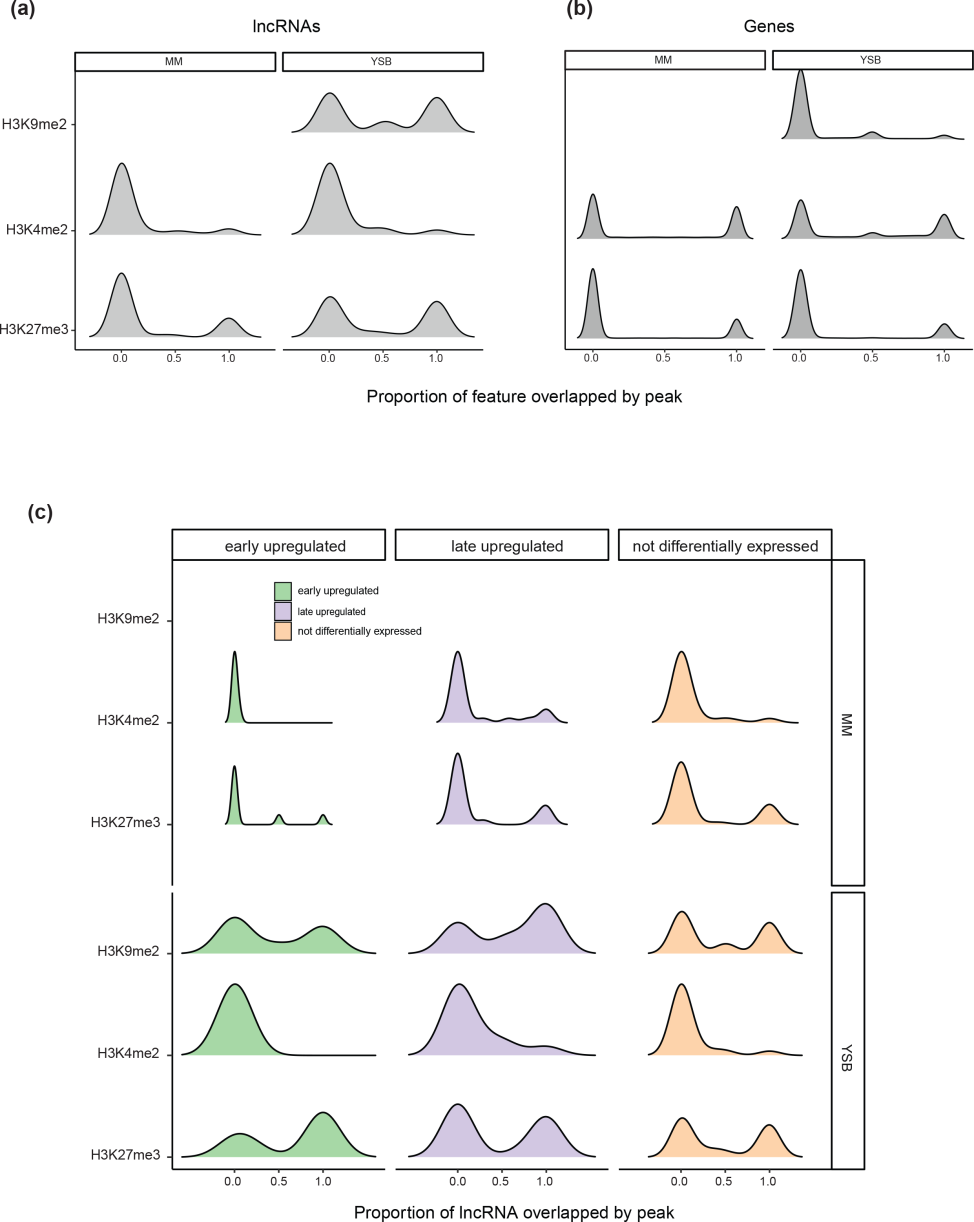
Density plot showing the coverage of (**a**) lncRNAs and (**b**) protein-coding genes according to different histone methylation markers in minimal media (MM) and yeast-sucrose broth (YSB). MM mimics conditions during early infection. The proportion of each locus covered by the marker is shown. (**c**) Density plot comparing the coverage of lncRNAs by different histone methylation marks in MM and YSB, based on the differential expression status of the lncRNAs and the time point of their peak expression.

In *Z. tritici,* genes encoding virulence factors are probably regulated through histone modification [98]. Notably, heterochromatin associated with H3K27me3 and H3K9me3 is enriched in both species-specific and biosynthetic genes, whereas euchromatin associated with H3K4me2 covers mostly gene-dense and conserved regions [98]. Data for H3K27me3, H3K9me3 and H3K4me2 [99] for the reference isolate IPO323 grown in both minimal media (MM), and H3K4me2 and H3K27me3 data for growth in YSB were analysed. Compared to genes, lncRNAs were less likely to be covered by H3K4me2 in both growth conditions (Welch two-sided *t*-tests; *P*<2.2e-16) (Fig. 7a, b). lncRNAs were more likely to be covered by H3K9me2 in YSB (Welch two-sided t-test, *P*=1.007e-10), and H3K27me3 in both MM and YSB (Welch two-sided t-test, *P*=2.585e-07) compared to genes (Fig. 7a, b). Interestingly, lncRNAs, but not protein-coding genes, showed an increase in H3K27m3 coverage in YSB compared to MM (Fig. 7a). The higher proportion of lncRNAs covered by H3K27me3 compared to protein-coding genes is consistent with the notion that lncRNAs are likely to be species-specific.

Soyer *et al.* [98] showed that *in vitro* H3K27me3 and H3K9me3 marked regions were enriched in genes that were differentially expressed during early host colonization or at the switch to necrotrophic growth. We compared *in vitro* chromatin profiles of lncRNAs up-regulated during early infection to those up-regulated during late infection. In MM, we find that early up-regulated lncRNAs lack coverage by H3K4me2 and are rarely covered by H3K27me3 (one out of 22 early up-regulated lncRNAs are fully covered by H3K27me3 in MM). In contrast, late up-regulated lncRNAs show most coverage by H3K7me3 (four out of eight late up-regulated lncRNAs are fully covered by H3K27me3 in MM) (Fig. 7c). In YSB, all lncRNAs show an increase in coverage by H3K27me3. The MM conditions *in vitro* resemble conditions during early growth *in planta,* due to the lack of external resources and higher levels of stress [49]. Hence, the lncRNAs involved in early infection may be epigenetically regulated and repressed by H3K27m3.

### Pangenome analyses of lncRNA diversity

**Fig. 8.**
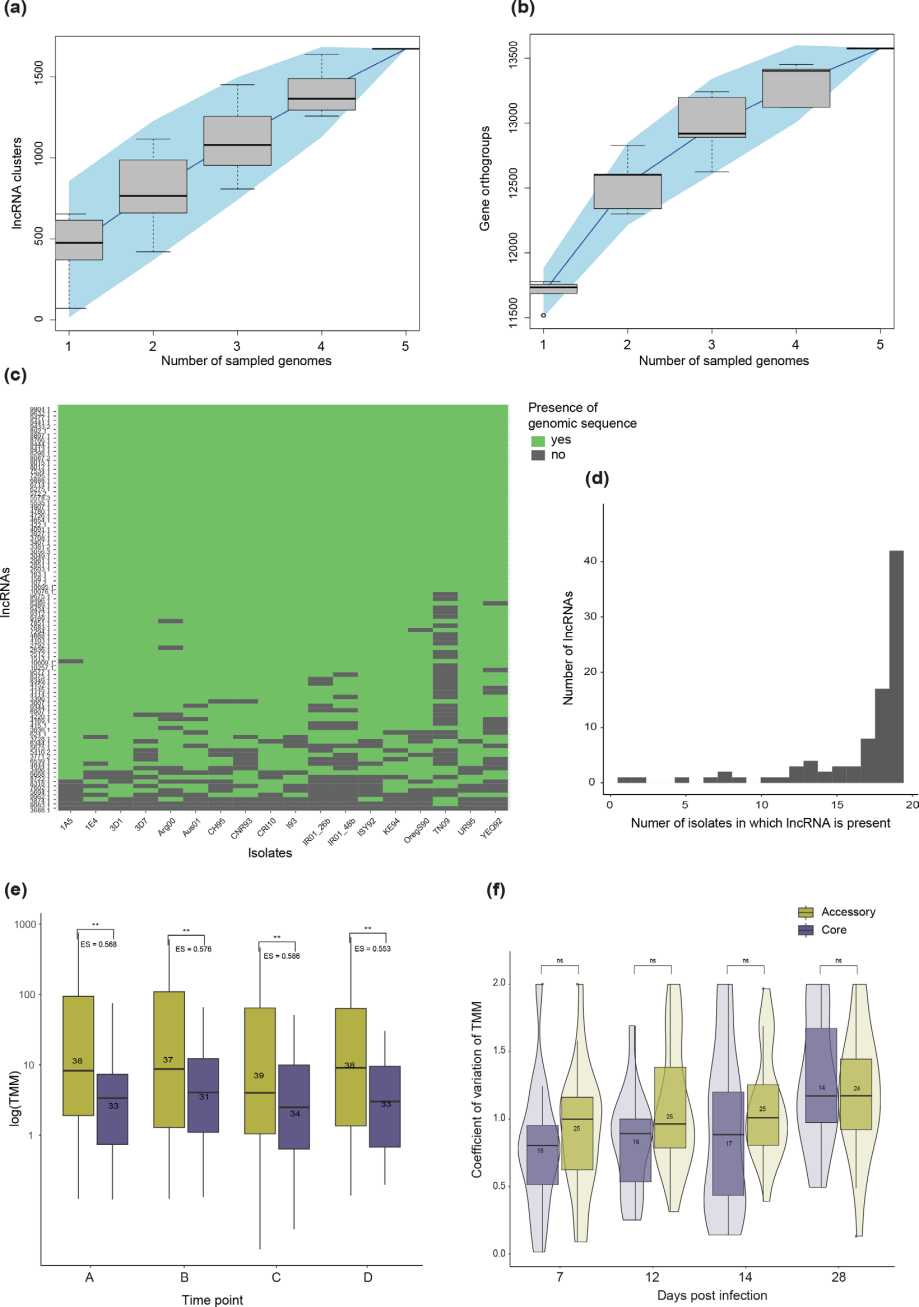
(a) Accumulation curve of lncRNAs predicted for four additional reference strains clustered based on sequence similarity. (**b**) Accumulation curve of gene orthogroups in the same reference strains. (**c**) Heatmap showing the presence of each original lncRNA (predicted in IPO323) in 19 different isolates at the genomic level. If genomic sequences are identified to be similar to the lncRNA reported in Zt09, the heatmap is coloured in green. (**d**) Bar plot showing the number of genomes in which the original lncRNAs (predicted in IPO323) returned a significant hit. (**e**) Comparison of expression values (TMM) between core (found in at least 18 genomes) and accessory lncRNAs (missing in at least one genome), for all original lncRNAs (predicted in IPO323) at different time points during the infection cycle. Stars represent the significance level of Welch's two-sided *t-*tests comparing mean expression of core and accessory lncRNAs. Values denoted by ‘ES=’ show the effect size relative to the expression of accessory lncRNAs. Numbers in each box show the sample sizes. (**f**) Violin plot comparing the coefficient of variation (CV) in expression between core and accessory lncRNAs, calculated by comparing centred and scaled TMM values between four isolates across the infection cycle. Numbers inside the boxes show the sample size in each group.

*Z. tritici* populations are highly diverse even at small geographical scales [42, 100, 101], and the species carries a vast accessory genome [61, 73, 102]. We aimed to understand if lncRNAs were as diverse as proteins across the pangenome of the species. To compare protein diversity to lncRNA diversity, we repeated our prediction pipeline using transcriptomic data from four additional isolates (1A5, 1E4, 3D1 and 3D7), collected at similar time points during the infection cycle as the original data for Zt09. We clustered all predicted lncRNA transcripts using relatively relaxed stringency criteria (sequence identity ≥80 %, minimum alignment coverage 75%) to account for the fact that lncRNA sequences may diverge rapidly [103]. We obtained 1671 clusters of lncRNAs, of which 1233 contained only a single transcript. We constructed an accumulation curve using the cluster attributions (Fig. 8a) and compared this to the accumulation of protein orthogroups which had previously been constructed for the same isolates [73] (Fig. 8b). Compared to proteins, the slope of the lncRNA accumulation curve was steep and without any indication of a plateau, suggesting that lncRNAs are more diverse. However, many lncRNAs show highly specific expression patterns and may only be expressed during particular stages of development and conditions [103]. Condition specificity is much higher than for protein-coding genes [26]. Considering that the data for 1A5, 1E4, 3D1 and 3D7 were taken at the same time points regardless of morphological characteristics, some of the observed diversity for lncRNAs may be attributable to small differences in developmental stage, even at identical time points.

To assess whether the observed lncRNA diversity is linked to expression variability, we performed a sequence-similarity-based search for each original lncRNA identified in Zt09 in 18 additional reference-quality genome assemblies, including the four used in the previous section (Fig. 8c). In contrast to the transcriptome-based approach, we found that the overwhelming majority of lncRNA sequences were conserved among the 19 genomes (Fig. 8d). Only a single lncRNA was found uniquely in Zt09. Hence, the pool of expressed lncRNAs probably varies greatly between strains, even if they are encoded in the genome of all individuals. We used genetic similarity to assign core (present in at least 18 out of 19 isolates) and accessory status for each lncRNA identified in Zt09. Core lncRNAs are expressed at significantly lower levels than accessory lncRNAs at all time points in Zt09 (average effect size of 0.570; Fig. 8e). Significant differences were also found in the strains 1A5, 1E4, 3D1 and 3D7, except at 7 dpi (Fig. S15). Low levels of lncRNA expression may be an important feature of lncRNA functionality, notably for those involved in chromatin organization [103, 104]. Compared to accessory lncRNAs, core lncRNAs tend to vary less in their expression between isolates, although these differences were not significant (Fig. 8f). Furthermore, core and accessory lncRNAs showed no distinct distribution (Chi-square test, *P*=0.24) among the original expression clusters (Fig. S16), even though accessory lncRNAs were underrepresented in the group of clusters showing peak expression at later time-points (Fig. S17).

## Discussion

We identified lncRNAs in the fungal pathogen *Z. tritici*, and evaluated potential regulatory and biological functions. Compared to similar genome-wide screens for lncRNA candidates in other organisms, including studies on other pathogenic fungi, our prediction yielded very low numbers of lncRNA candidates. Most studies report 1000–10 000 s lncRNAs per species [20, 31, 35, 37, 105]. We hypothesize that this results from a high number of false negatives. We chose to use a higher weight on the Youden’s index than the default parameter, which is used in the prediction tool to determine a cut-off for sensitivity. A higher weight improves false-positive rates but increases false-negatives [53]. Additionally, only a single transcript per locus was considered, despite the fact that lncRNAs can undergo alternative splicing [19, 106, 107]. The choice to include TE consensus sequences in the known set of mRNAs may have also increased false negatives considering that many lncRNAs may contain TE-associated elements [108]. Lastly, some lncRNAs may have been missed due to the single-ended nature of our RNAseq data, considering that paired-end reads increase library complexity and result in higher read counts per locus, particularly in non-coding regions, which improves transcript assembly [109]. Because we focused on *in planta* expression, a low proportion of the sequenced biological material originates from the pathogen, which results in low read counts. In such cases, the increased library complexity offered by paired-end sequencing may be of particular importance. Paired-end data can also improve the detection of overlapping same-sense transcripts [110], which may be the case for a substantial fraction of fungal lncRNAs. In *M. robertsii,* 12 % of identified lncRNAs share exons with known mRNAs [31].

Although many fungal lncRNAs are intergenic [31, 35, 37], evidence from several fungal pathogens suggests that a large proportion of their lncRNAs could be antisense to protein-coding genes [35, 38, 111]. Surprisingly, only two antisense lncRNAs were identified using our approach. Comparison with the new annotation of the reference genome [52], in which similar numbers of sense and antisense lncRNAs were found, suggests that technical issues are the most likely cause of the lack of antisense lncRNA in our predictions. In some cases, reads originating from the loci in the new annotation are present but at low depth, and/or they do not cover the full length of the transcript [52]. As such, transcripts were not properly assembled and were never assessed by the prediction tool. In other cases, transcripts were assembled correctly but were predicted as coding, perhaps because of shared features with the opposing coding sequence, which may make antisense lncRNA more challenging to distinguish from mRNA compared to intergenic lncRNA.

lncRNAs showed differences in epigenetic profiles compared to genes, and were more likely to be located in facultative heterochromatic regions. In *Z. tritici*, these regions are associated with effectors and biosynthetic genes, which are often up-regulated during early colonization [98]. The presence of lncRNAs in facultative heterochromatic regions may be indicative of their co-regulation with genes found in the same regions, which is consistent with the rest of the observations in this study. The fact that lncRNAs with peak expression during late infection were more likely to be covered by repressive markers in minimal media than those with peak expression at earlier times points suggests that lncRNA expression and chromatin state are linked. lncRNAs are known to contribute to the formation of heterochromatin in *Drosophila* and plants through interactions with chromatin-modifying enzymes [112]. A candidate lncRNA shows the potential to interact with HMT1, through a domain in a neighbouring, anti-correlated and potentially chemically targeted mRNA. The interaction is unlikely to be directly related to the formation of heterochromatin itself, but it demonstrates the potential for lncRNAs to be involved in chromatin dynamics in this species. As little is known about the mechanisms of epigenetic regulation in *Z. tritici,* it may be interesting for future research to consider the role of lncRNAs, especially in dynamically regulated regions.

lncRNAs were expressed at lower levels than mRNAs, which is consistent lncRNA expression in other organisms. As expected, expression is also generally more variable between isolates, except at 7 dpi. It is important to note that this estimate is susceptible to overestimation due to low read counts and high stochasticity. We find no evidence that the lncRNA position relative to a gene influences the level of co-expression between the pair, showing that lncRNAs are neither predominantly *cis*- nor *trans*-acting in this species. The potential for lncRNAs to form RNA-complexes with nearby mRNAs does significantly impact anti-correlation between lncRNA and mRNA expression at close distances. This suggests that lncRNA–mRNA interactions *in cis* may be one mechanism of lncRNA-mediated regulation. The majority of identified lncRNAs were up-regulated during early stages of host infection. Compared to genes, a significantly larger proportion of lncRNAs show expression profiles with peaks either before or during the switch to necrotrophic growth. Moreover, their location in specific chromatin regions is consistent with that of protein-coding genes expressed at these time points. Interestingly, this mirrors observations from the human microbial parasite *Cryptosporidium parvum* with lncRNAs being highly expressed during invasion and less during proliferation in contrast to mRNAs [33]. During early colonization, the pathogen must rely on internal nutrient stores, as the organism is unable to acquire these directly from the host [86]. The pathogen must also protect itself from host-defence mechanisms, such as the production of reactive oxygen species and chitinases [90, 113]. lncRNAs are known to regulate a wide array of stress-response pathways and changes in nutrient acquisition in other organisms [21, 34]. Hence, lncRNAs may be of particular importance during this life cycle stage. The idea that lncRNAs are involved in responding to early-infection stress in *Z. tritici* is supported by enriched functions among potential lncRNA targets, such as antioxidant or serine-hydrolase activity.

*Z. tritici* is a highly polymorphic species including at the level of gene presence–absence variation and accessory chromosomes [73]. We explored pangenomic variation of lncRNAs in comparison to genes. Most lncRNAs were located on the core chromosomes shared between all individuals of the species. Nevertheless, based on the transcriptomes of four reference isolates, we observe a high level of variation in lncRNA repertoires, and a steep accumulation curve compared to genes. At the genomic level, the majority of lncRNAs identified in the reference strain IPO323 are shared among 18 strains from around the world. Differences in the genomic and transcriptomic evidence of lncRNAs are best explained by the high specificity of lncRNAs [26]. lncRNA expression is highly dependent on environmental conditions, and small changes in developmental stage of either the pathogen or the host, or in experimental conditions, may result in vastly different lncRNA repertoires being expressed. While similar loci are shared among isolates, mutations may have accumulated in these loci to affect how lncRNAs are transcribed. Future studies should aim to disentangle transcriptomic diversity from genomic diversity, particularly in the context of lncRNAs. In conclusion, our study provides a repertoire of lncRNA loci for *Z. tritici* and how these probably intervene in host infection processes and the responses to stress.

## Supplementary Data

Supplementary material 1

Supplementary material 2
